# The Mechanical Properties and Biometrical Effect of 3D Preformed Titanium Membrane for Guided Bone Regeneration on Alveolar Bone Defect

**DOI:** 10.1155/2017/7102123

**Published:** 2017-09-05

**Authors:** So-Hyoun Lee, Jong-Hoon Moon, Chang-Mo Jeong, Eun-Bin Bae, Chung-Eun Park, Gye-Rok Jeon, Jin-Ju Lee, Young-Chan Jeon, Jung-Bo Huh

**Affiliations:** ^1^Department of Prosthodontics, Dental Research Institute, Institute of Translational Dental Sciences, BK21 PLUS Project, School of Dentistry, Pusan National University, Yangsan 50612, Republic of Korea; ^2^Biomedical Engineering, School of Medicine, Pusan National University, Yangsan, Republic of Korea; ^3^School of Dentistry, Pusan National University, Yangsan, Republic of Korea

## Abstract

The purpose of this study is to evaluate the effect of three-dimensional preformed titanium membrane (3D-PFTM) to enhance mechanical properties and ability of bone regeneration on the peri-implant bone defect. 3D-PFTMs by new mechanically compressive molding technology and manually shaped- (MS-) PFTMs by hand manipulation were applied in artificial peri-implant bone defect model for static compressive load test and cyclic fatigue load test. In 12 implants installed in the mandibular of three beagle dogs, six 3D-PFTMs, and six collagen membranes (CM) randomly were applied to 2.5 mm peri-implant buccal bone defect with particulate bone graft materials for guided bone regeneration (GBR). The 3D-PFTM group showed about 7.4 times higher mechanical stiffness and 5 times higher fatigue resistance than the MS-PFTM group. The levels of the new bone area (NBA, %), the bone-to-implant contact (BIC, %), distance from the new bone to the old bone (NB-OB, %), and distance from the osseointegration to the old bone (OI-OB, %) were significantly higher in the 3D-PFTM group than the CM group (*p* < .001). It was verified that the 3D-PFTM increased mechanical properties which were effective in supporting the space maintenance ability and stabilizing the particulate bone grafts, which led to highly efficient bone regeneration.

## 1. Introduction

The sufficient residual alveolar bone volume is the critical factor to determine the long-term survival and success of the dental implant treatments [1]. Alveolar bone defects of the intended implant placement site result from infection, trauma, and lesion and numerous procedures including block bone grafting [2], ridge splitting [3], distraction osteogenesis [4], and guided bone regeneration (GBR) [5] are introduced for the bone reconstruction. GBR is a surgical procedure that uses barrier membrane at the bone defected site to block the migration of epithelial cells and connective tissues and enhance the osteogenesis by stabilizing blood clot and bone-forming cells [5]. In particular, Buser et al. [6] pointed out that the use of barrier membrane with insufficient rigidity and space maintenance in the bone defects causes displacement of the grafts from the stress in the oral soft tissues, resulting in inadequate bone regeneration [7].

The barrier membranes used in GBR procedure have two different types, resorbable and nonresorbable [8–10]. The resorbable barrier membrane such as collagen membrane is commonly preferred in the clinical field since it has good biocompatibility and eliminates the second surgery to remove the membrane [11]. However, its physical properties reduced when it is exposed to blood or water and space-making ability lacked once the implant is placed and sutured [12]. On the other hand, nonresorbable barrier membrane such as expanded polytetrafluoroethylene (e-PTFE) and titanium- (Ti-) mesh has excellent mechanical strength and space-making ability and it has been selectively used in surgical procedure that requires large amount of bone graft materials [13, 14].

Since the introduction of the Ti-mesh in 1969 by Boyne et al. [15], it has been widely used in various fields including GBR, maxillofacial fracture, and reconstruction [16, 17]. Ti-mesh has high strength and rigidity, low density, plasticity, and corresponding low weight [18]. Moreover, it has the ability to withstand high temperatures and its resistance to corrosion [19–22]. Although many studies evaluated and verified the efficacy of Ti-mesh as barrier membrane in stabilization of bone materials [15–22], osseous space maintenance ability, and bone regeneration [23–25], there are some major disadvantages that limit its application in GBR [13]. Her et al. [22] indicated that additional surgery procedures for Ti-mesh removal are the main disadvantages of long-term healing periods and additional mucosal injury [14]. Louis et al. [26] mentioned that the other problem is time-consuming manipulation process such as cut, trimming, and bending the Ti-mesh plate to fit various bone defects [22]. In addition, Becker et al. [27] reported that sharp edges or surfaces of Ti-mesh during the manipulation lead to mechanical irritation in mucosal flap and finally may result in Ti-mesh exposure [28].

Recently, the preformed titanium membrane (PFTM) has been introduced to overcome these disadvantages of conventional Ti-mesh. Conventional Ti-mesh was uniformly fabricated as a two-dimensional (2D) plate without consideration of the bone defect types [26, 29, 30]. On the other hand, PFTM has been designed and manufactured in various forms of bone defects commonly observed clinically, and the operator can minimize manual procedures to apply at GBR [31]. The PFTM system is composed of the anchor, cover cap, and PFTM. For the clinical uses, a PFTM is selected according to the size and shape of peri-implant bone defect and is placed on the anchor connected to implant fixture and cover cap is applied on top for direct fixation between implant and PFTM [6]. The selection of various designed PFTM instead of manipulation process of conventional Ti-mesh was expected to improve the clinical manageability, minimize membrane exposure, and increase peri-implant alveolar bone regeneration [32]. However, existing PFTM is still necessary for additional manually shaped process such as bending [28].

Therefore, in this study, three-dimensionally preformed titanium membrane (3D-PFTM) developed by new mechanically compressive molding technology was used [32]. There have been few studies on the effect of this new technique on the mechanical properties and biological stability of PFTM to be used in GBR. The purpose of present study was to compare the mechanical properties of the 3D-PFTM with the manually shaped preformed titanium membrane (MS-PFTM) through the artificial peri-implant bone defect model and to compare the biological stability of the 3D-PFTM with the collagen membrane through the large animal peri-implant bone defect model.

## 2. Materials and Methods

### 2.1. Fabrication of the 3D Preformed Ti-Membranes (3D-PFTMs) and Manually Shaped Ti-Membranes (MS-PFTMs)

**Figure 1:**
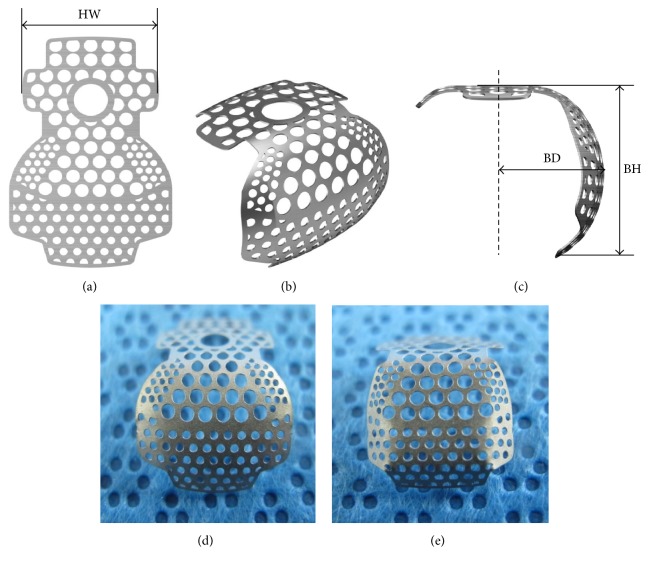
Designs of Ti-membranes. (a) Preformed Ti-membrane (PFTM) designed for the peri-implant buccal bone defect site. (b) Buccal view of deformed PFTM. (c) Lateral view of deformed PFTM (HW, horizontal width; BH, buccal height; BD, buccal depth). (d) 3D preformed Ti-membranes (3D-PFTMs). (e) Manually shaped Ti-membranes (MS-PFTMs).

Ten Ti-membranes of plate form were prepared for this study. The Ti-membranes were preformed for buccal bone defect sites around implants and were deformed using hand or mechanical methods (Figure 1). In the experimental group, five preformed Ti-membranes (PFTMs) were transformed into 3D-PFTM (SmartBuilder^TM^ SM3W10129SB, Osstem Implant Co., Seoul, Korea) of semi-dorm shape using the press molding machine (SBPM1004, Seoul, Korea) (Figure 1(d)). In the control group, five PFTMs were transformed into MS-PFTM by hands (Figure 1(e)). All Ti-membranes were 0.1 mm in thickness and were designed to be sufficient size (horizontal width (HW): 10 mm, buccal height (BH): 7 mm, and buccal depth (BD): 5.5 mm) to completely cover the buccal bone defects around implants. Ti-membranes were fabricated with three different pore sizes. The central pore size is 1.0 mm for blood supply and marginal pore size is 0.6 mm and 0.5 mm for lateral tissue integration [33].

### 2.2. In Vitro Study for Mechanical Properties of 3D-PFTM

#### 2.2.1. The Static Compressive Load Test

**Figure 2:**
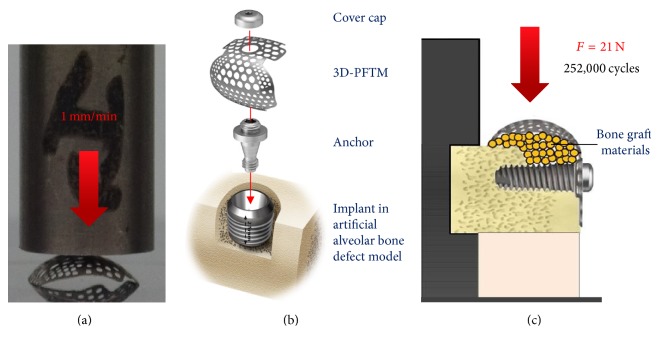
Schematic diagrams of measurements for mechanical properties. (a) The static compressive load test. (b) Assembly of 3D-PFTM or MS-PFTM consisting of PFTM, anchor, and cover cap. (c) The cyclic fatigue load test.

To compare the mechanical stiffness of the 3D-PFTM and the MS-PFTM, a static test was conducted using a universal compression and tension testing machine (Instron E3000 ElectroPlus, Mass, Norwood, MA, USA). Five specimens were used for each group and the jig was placed close to the center of the buccal surface of the specimen. The compressive load was vertically applied at a rate of 1 mm/min and the primary plastic deformation of the specimen was measured (Figure 2(a)).

#### 2.2.2. The Cyclic Fatigue Load Test

A fatigue test was conducted using a hydraulic vertical load machine (Instron 8841 DynaMight™, Mass, Norwood, MA, USA) to simulate deformation of 3D-PFTM and the MS-PFTM after cyclic fatigue loading by intraoral soft tissue.

For this experiment, the alveolar bone defect model was fabricated using artificial bone material (cortical bone # 40, cancellous bone # 20, and saw bone, WA, USA). After forming a 2.5 mm height buccal bone defect, the implant (TSIII, Ø3.5 × H7.0 mm, Osstem Implant Co., Seoul, Korea) was placed with a force of 30 Ncm until the platform of the implant fixation was located 1 mm below the cortical bone of the model. The anchor was connected to implant fixture and was tightened to torque of 8 Ncm, using a digital torque gauge (MGT12E, Hicksville, NY, USA). To simulate the GBR process, approximately 0.1 mg of bone graft materials (A-Oss, Osstem Implant Co., Seoul, Korea) was used to fill the buccal defects. The 3D-PFTM or the MS-PFTM was placed on top of the anchor and fixed to the implant using a cover cap with a force of 13 Ncm (Figure 2(b)). The jig was fabricated using soft melting and elastic impression material (Hyflex, Osstem Implant Co., Seoul, Korea) to stimulate the cyclic fatigue loading.

The cyclic fatigue load was set as 21 N [34] to demonstrate the maximum force of intraoral soft tissue and it was applied twice per one second with the speed of 2 Hz on the buccal direction of Ti-membrane. A total of 252,000 cycles were applied considering 1,400 cycles, the maximum number of chewing per day [35], and the clinically proven 6 months of osteogenesis [36]. The vertical distance between the Ti-membrane buccal side and the floor was 18.3 cm (Figure 2(c)). The structural changes and vertical distance were measured after cyclic fatigues were loaded.

### 2.3. In Vivo Study for Biometric Effects of 3D-PFTM

#### 2.3.1. Experiment Animals

The space maintenance ability, biological compatibility, and effectiveness in bone regeneration of PFTM were studied in three healthy male beagle dogs, 18 months of age and weighing approximately 10 kg. This in vivo experiment was checked with the modified ARRIVE guidelines [37, 38]. The animal selection, management, and surgical protocols were previously reviewed and approved by the Ethics Committee on Animal Experimentation of Chonnam National University (CNUIACUC-TB-2013-10). The premolars and first molars from the bilateral mandible were extracted in the first surgery. Teeth extraction was carefully proceeded to preserve the buccal, lingual, and lateral walls of the alveolar sockets and no damage was found at the extraction site. The extraction sites of animals were sutured using 4-0 nylon (Mersilk, Livingston, UK) to enhance healing and left eight weeks to heal completely.

#### 2.3.2. Implant Surgery and Guided Bone Regeneration Procedures

**Figure 3:**
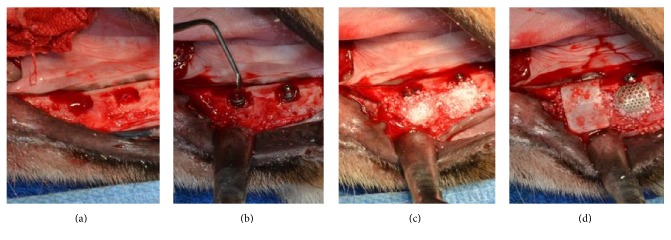
Implant surgery and guided bone regeneration procedures for the in vivo study. (a) The buccal open defects 2.5 mm were formed on the mandible of the experiment animal. (b) The implants were placed on the buccal open defects. (c) All the defects were filled with the particulate bone graft materials. (d) The collagen membrane (CM) and 3D preformed Ti-membranes (3D-PFTMs) were placed randomly on the buccal open defects. The 3D-PFTM connected to implant fixture with anchor and cover cap.

The implant surgery and GBR were processed after the extraction sockets were completely healed. Before the implant placement, the alveolar ridge was reduced down to make it flat and 2 peri-implant buccal bone defects were formed on unilateral mandible of each dog (4 defects per animal) (Figure 3(a)). The distance between the implant placement sites was measured using a ruler and the position was marked to constantly place the implant in bilateral mandible. Each of the 12 implants (TSIII, Ø3.5 × H7.0 mm, Seoul, Korea) was placed on 12 peri-implant buccal bone defects formed in three dogs. Lower 4.5 mm part of implant was inserted at the flattened alveolar ridge and approximately upper 2.5 mm part of implant exposed at the peri-implant buccal bone defects (Figure 3(b)). The 0.1 mg deproteinized bovine bone graft materials (Bio-Oss, Wolhusen, Switzerland) were applied to each peri-implant buccal bone defect (Figure 3(c)). Subsequently, six collagen membranes (GENOSS, Suwon, Korea) and six 3D-PFTM (SmartBuilder, Seoul, Korea) were randomly inserted on the peri-implant buccal bone defect areas (Figure 3(d)). For the membrane connection of each implant, a height connector (SmartBuilder SB Anchor for TS, Ø4.0 × H0.5 mm, Seoul, Korea) and the cover cap (cover cap, Ø4.0 × H1.5 mm, Seoul, Korea) were inserted for the fixture and stabilization of the membrane. The mucoperiosteal flaps were advanced, adapted, and sutured to submerge the implants.

#### 2.3.3. Postoperative Care and Sacrifice

Antibiotics, penicillin G procaine and penicillin G benzathine, were given via intramuscular injection (1 mL/5 kg) immediately after the surgery and 48 hours after the surgery. 2% chlorhexidine gluconate was used to control plaque by flushing the oral cavity daily until the end of the study. The soft chewable foods were given for two weeks and the regular diet was provided through the study. Animals were sacrificed using intravenous injection of concentrated sodium pentobarbital (Euthasol, Midlothian, VA, USA) eight weeks after the surgery. The study specimens, including the alveolar bones near the implant, membranes, and surrounding mucosae, were obtained from the mandibles of the sacrificed beagles. Neutral buffered formalin (Sigma Aldrich, St. Louis, MO, USA) was used to fix the harvested mandible specimens for two weeks and dehydrated in numerous concentrations of ethanol from 70% to 100%.

#### 2.3.4. Histomorphometric Analysis

**Figure 4:**
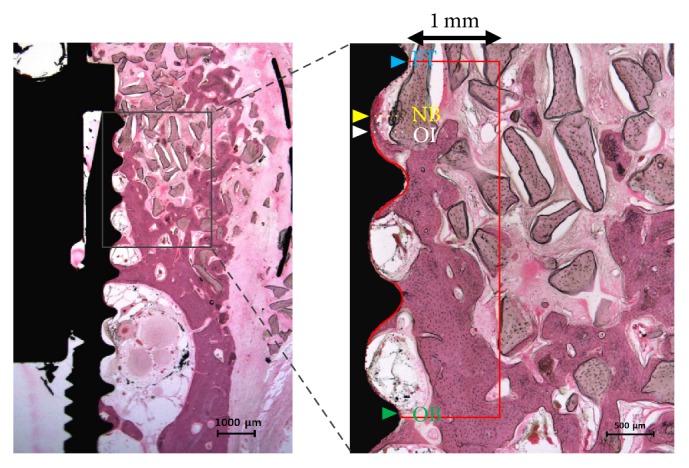
Parameters measured in the histologic specimens. Red box, the area of interest (AOI was 1 mm horizontally and vertically a range from the horizontal crest of the first thread of the implant to the horizontal crest of the third thread); blue arrow, the horizontal crest of the first thread of the implant (FT); yellow arrow, the most upper point of the new bone (NB); white arrow, the most upper point of the osseointegration site (OI); green arrow, the most upper point of the old bone of AOI (OB).

Ethanol, increasing concentration up to 100%, was used to cleanse and dehydrate the specimens and infiltration occurred with the increasing Technovit 7200 resin (Heraeus Kulzer, Germany) to ethanol ratio. Subsequently, the specimen was inserted and fixed on the embedding frame and put into a UV embedding system (Kulzer Exakt 520, Germany) for a day to cure the resin. The EXACT diamond cutting system (EXACT 300 CP, Germany) was used to slide the polymerized specimen block longitudinally at the implant center and the adhesive press system was used to attach the block to slide. The EXACT grinding system (Kulzer EXACT 400CS, Germany) was applied to grind the block within the range of thickness from 400 *μ*m to 40 ± 5 *μ*m. The hematoxylin and eosin staining (H&E staining) was applied, before mounting the sample, to observe the newly regenerated bone tissues in the specimen. Subsequently, the final slides were prepared and their images were captured using the CCD camera (Spot Insight 2 Mp scientific CCD digital camera system, USA) and adaptor (U-CMA3, Olympus, Japan) that were mounted on the light microscope (BX51, Olympus, Japan). i-Solution version 8.1 (IMT i-Solution Inc., Coquitlam, BC, Canada) was applied to analyze the captured images of the specimens. The captured images at 12.5x magnification were used for the general analysis and the histometric analyses were conducted at 40x magnification. A single examiner, professionally trained and blinded to the specimen groups, performed the histometric analyses to conduct the following measurements within the area of interest (AOI). The area of interest (AOI) is 1 mm horizontally and is vertically a range from the horizontal crest of the first thread of the implant to the horizontal crest of third thread (Figure 4).

The following measurements were analyzed and recorded within the area of interest (AOI).

- New bone area (NBA; %): area occupied by the new bone/AOI × 100
- Remaining bone substitute area (RBA; %): area occupied by the remaining bone substitute/AOI × 100
- Bone-to-implant contact (BIC; %): length of the contact with the new bone/total length of the exposed threads × 100
- New bone-old bone (NB-OB; %): distance from the most upper point of the new bone to the most upper point of the old bone/FT-OB × 100
- Osseointegration-old bone (OI-OB; %): distance from the most upper point of the osseointegration to the most upper point of the old bone/FT-OB × 100

#### 2.3.5. Statistical Analysis

The experimental data were expressed as means, standard deviations, and medians and the statistical analysis was performed using software R (version 3.1.3). Two types of membranes were set as an independent factor and the number of dogs was set as a random factor. The nonparametric mixed model was used to compare the radiographic and histomorphometric parameters and the post hoc analyses were conducted [39]. Statistical significance was at 5% level.

## 3. Results

### 3.1. Mechanical Properties Analysis

**Figure 5:**
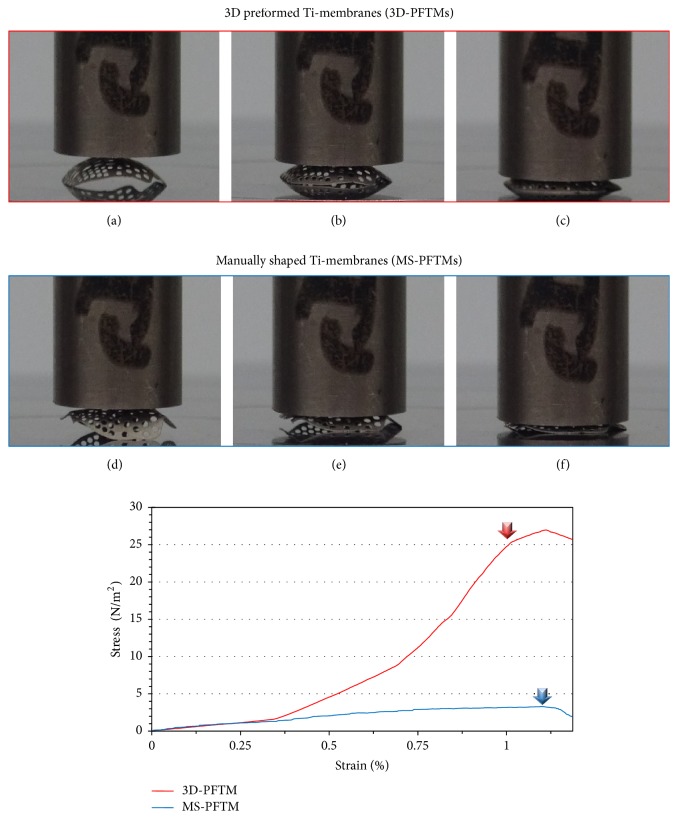
Deformation photographs and stress-strain graphs after static compressive load test. In the 3D-PFTM group, (a) initial state, (b) compressive loading, and (c) primary plastic deformation occurred. In the MS-PFTM group, (d) initial state, (e) compressive loading, and (f) primary plastic deformation occurred. The red (3D-PFTM) and blue (MS-PFTM) arrows in the graph indicated the load at the primary plastic deformation.

The results of the static compressive load test are shown in Figure 5. The primary plastic deformation of the 3D-PFTM group presented at 25.1 ± 0.44 N/m^2^ and that of the MS-PFTM group at 3.2 ± 0.12 N/m^2^. The mechanical stiffness of 3D-PFTM was confirmed to be about 7.4 times higher than that of MS-PFTM (Figure 5).

**Figure 6:**
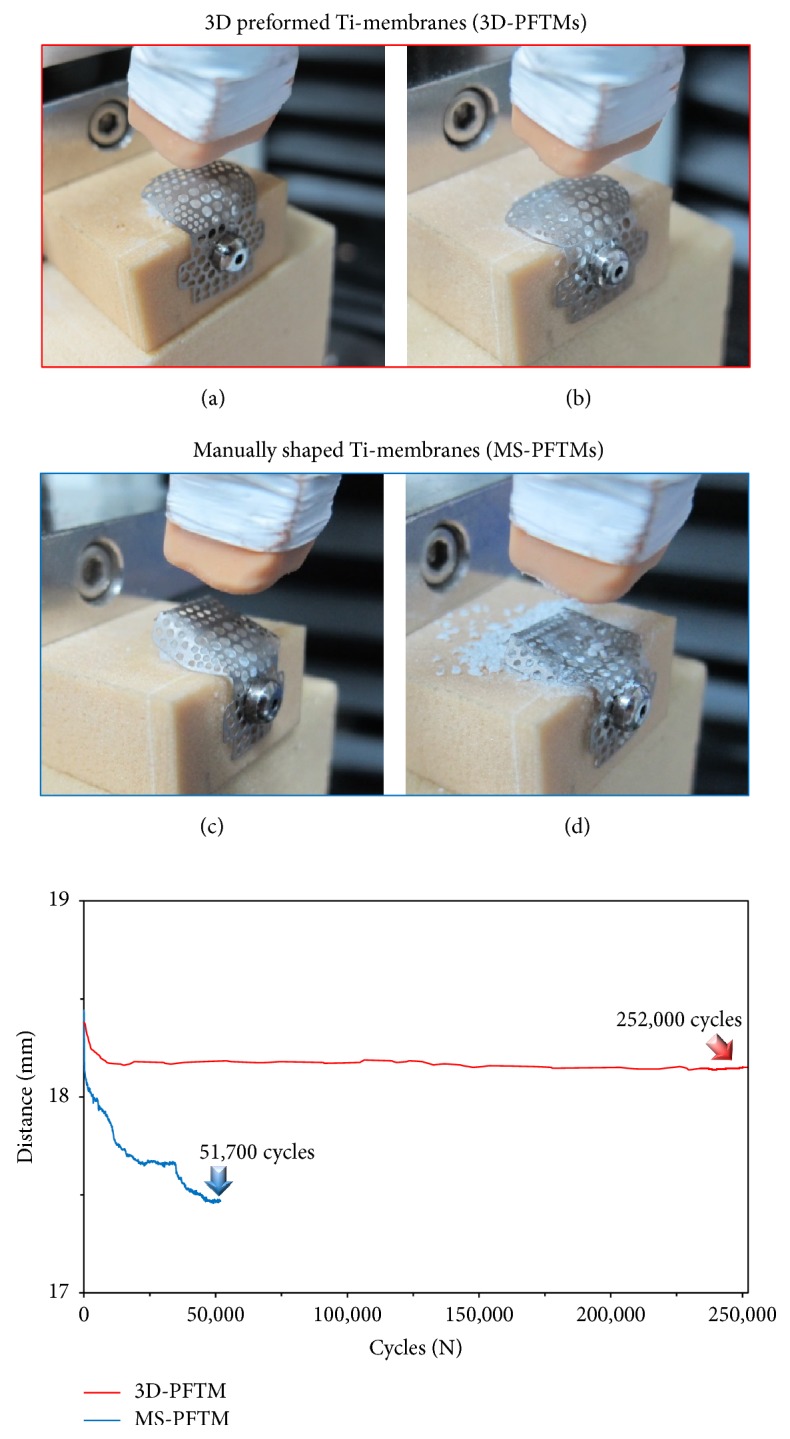
Deformation photographs and distance-cycles graphs after cyclic fatigue load test. (a) The initial state of 3D-PFTM covering bone grafts filled with artificial bone defects. (b) The original shape of 3D-PFTM was retained after 252,000-cycle fatigue load test (red arrow in the graph). (c) The initial state of 3D-PFTM covering bone grafts filled with artificial bone defects. (d) The original shape of MS-PFTM severely deformed after 51,700-cycle fatigue load test (blue arrow in the graph).

The results of the cyclic fatigue load test are shown in Figure 6. After 252,000-cycle fatigue loading, the buccal position of 3D-PFTM from floor was vertically reduced from 18.3 mm to 18.15 mm (Δ = 0.15 mm). The original form of 3D-PFTM was retained and the bone grafts were maintained without loss. On the other hand, the MS-PFTM was vertically reduced from 18.3 mm to 17.3 mm (Δ = 1.0 mm) after 51,700-cycle fatigue loading. The center of the MS-PFTM was pressed, and the lateral and inferior sides were flattened, resulting in the scattering and loss of bone grafts.

### 3.2. Clinical Findings

All experimental animals survived through the surgical procedures, and the 12 implant sites healed without inflammatory reaction. The membranes exposure did not occur during the healing period and implant failure was not observed.

### 3.3. Histologic Analysis

**Figure 7:**
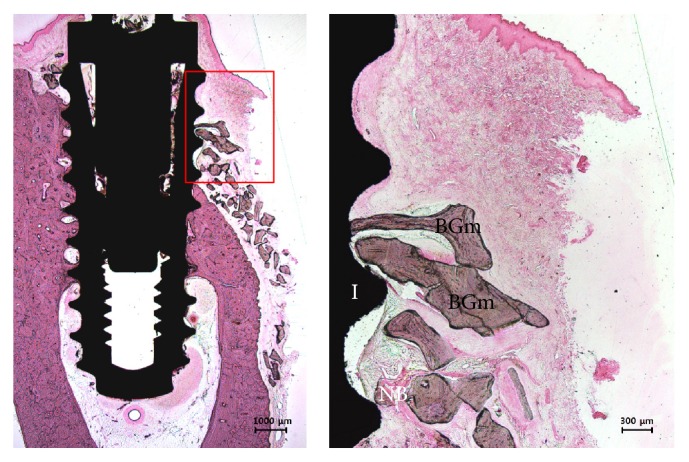
The histological images of collagen membrane (CM) group. No membrane was observed in some specimens and the particulate bone graft materials were scattered to bone defect site in peri-implant. Amount of new bone tissues and osseointegration were less. NB, new bone; BGm, bone grafting material; I, implant (H&E stain; magnification 12.5x [left] and 40x [right]).

**Figure 8:**
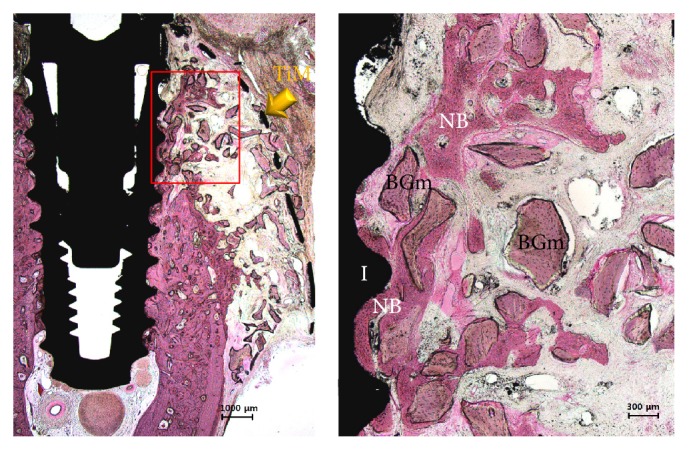
The histological images of 3D-PFTM group. All membrane and the more amounts of the particulate bone graft materials were observed on the bone defect site in peri-implant. New bone formation and osseointegration occurred. NB, new bone; BGm, bone grafting material; I, implant; TiM, Ti-membrane (H&E stain; magnification 12.5x [left] and 40x [right]).

During healing periods, there was no failure of implants and exposure of membranes. In some specimens of control group, no membrane was observed due to complete absorption and fibrous tissues were observed. The particulate bone graft materials were scattered to buccal bone defects of peri-implant and it was barely observed at the most upper thread (Figure 7). Compared with the control group, it was observed that large amounts of new bone tissue and particulate bone graft materials remained in the 3D-PFTM group. All Ti-membranes of the 3D-PFTM group remained and the original semi-dorm shape was well preserved. New bone formation mainly occurred around the bone graft materials and old bone. Osseointegration was observed along the threads (Figure 8).

### 3.4. Histometric Analysis

**Figure 9:**
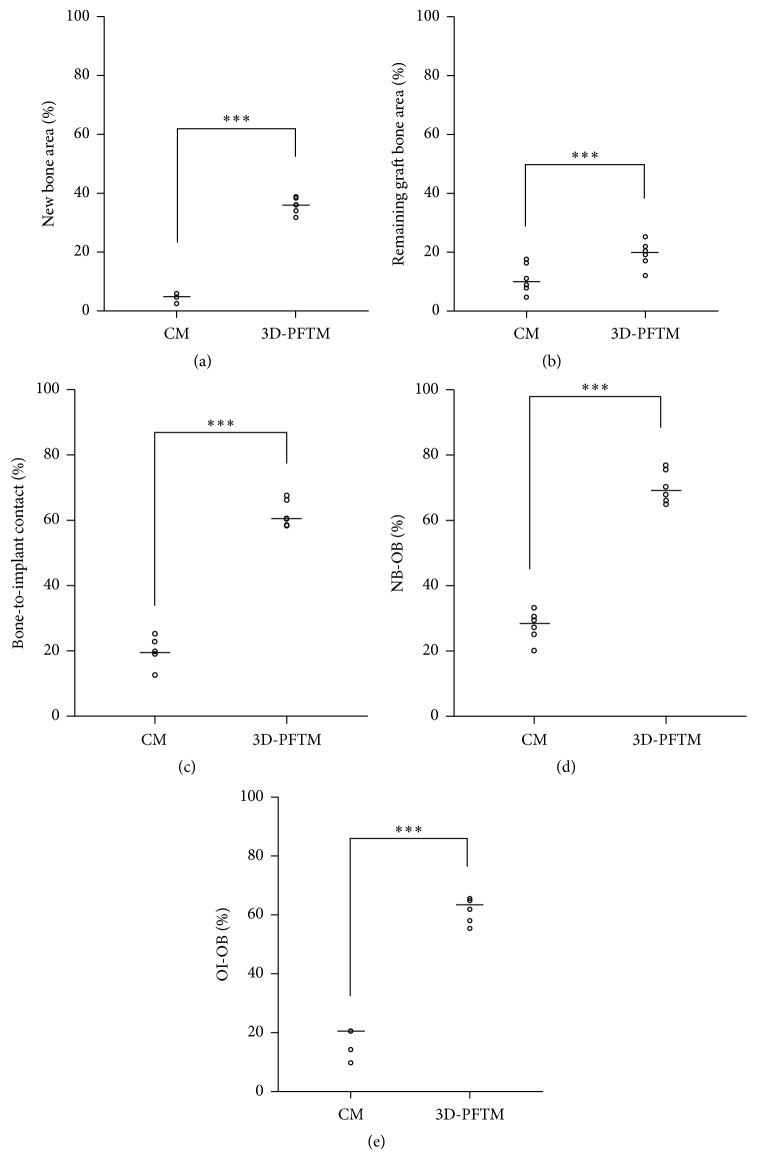
Scatter plot and median (the cross) representing graph of the control group (CM) and experimental group (3D-PFTM): (a) the area of newly formed bone tissues (NBA), (b) the remaining area covered by the bone graft substitutes (RBA), (c) bone-to-implant contact within the defect (BIC), (d) distance from the new bone to the old bone (NB-OB), and (e) distance from the osseointegration to the old bone (OI-OB) (*n* = 6). ^*∗∗∗*^Significantly different (*p* < .001).

The histometric measurements are summarized in Table 1. The remaining graft bone area (RBA, %) of the 3D-PFTM groups was greater than that of the control group (*p* < .001). The stabilization of particulate bone graft materials and space maintenance ability of 3D-PFTM were confirmed. Furthermore, the 3D-PFTM groups had significantly higher levels in the results of the new bone area (NBA, %), the bone-to-implant contact (BIC, %), distance from the upper point of new bone to the old bone (NB-OB, %), and distance from the upper point of the osseointegration to the old bone (OI-OB, %) compared to the control group (*p* < .001) (Figure 9).

## 4. Discussion

Many studies suggested using Ti-membrane with particulate bone graft materials in GBR [26, 40], because this combination creates a better resistance shield against the collapse of soft tissues and highly predictable space maintenance ability compared to other membranes [41–44]. Traditional Ti-mesh of two-dimensional (2D) plate form has been used to completely cover the bone defects site filled with bone graft materials through manual manipulation [15–22]. Recently, it has been advanced to fabricate customized bone defect form and directly connect it over the implant fixture [31–33]. The three-dimensional preformed Ti-membrane (3D-PFTM) used in this study was designed in consideration of the buccal bone defects that are mainly observed in the clinic, and it was prefabricated in 3D shape with new processing technology using a press molding machine.

Mechanical properties analysis confirms that the 3D-PFTM has sufficient rigidity and effective space maintenance ability to prevent deformation due to external stress without displacement of the particulate graft materials filled in bone defect area compared to MS-PFTM. The static compressive load test was conducted to verify the mechanical rigidity of 3D-PFTM produced by the new molding technology. According to the static compressive load test result, the primary plastic deformation was 25.1 ± 0.44 N/m^2^ for 3D-PFTM and 3.2 ± 0.12 N/m^2^ for MS-PFTM and the mechanical stiffness of 3D-PFTM was about 7.4 times higher than that of MS-PFTM. While the 3D-PFTM was pressed only at the center of the membrane, more strain was observed in the MS-PFTM where the sides and underside of the wing portion of the membrane were flattened. The cyclic fatigue loading test was conducted to evaluate stabilization of the particulate bone graft materials and space maintenance ability of 3D-PFTM in the oral cavity condition. To demonstrate the clinical stress due to the soft tissues of the GBR site, the cyclic fatigue load was set at 21 N, the maximum force of the soft tissue in the oral cavity, and repeatedly tested at 252,000 cycles, the maximum number of chewing instances during the six months of bone regeneration [34–36]. After all preplanned cyclic fatigue loads of 252,000 cycles were applied, the 3D-PFTM kept the original shape consistent and was maintained without loss of bone graft material. On the other hand, the MS-PFTM occurred after 51,700 cycles of irreversible deformation and lost its ability to maintain space, failing further experiments.

The thickness of barrier membrane for GBR is one of the important factors that affect the space maintenance and collapse of soft tissue; thus the adequate thickness of membrane is needed within the range that does not induce mucosal irritation [9]. As the membrane thickness increases, the mechanical stiffness and space maintenance ability are improved while clinical manageability is reduced. As the membrane thickness decreases, the stiffness becomes weak and eventually loses its space maintenance ability due to the external force [43]. The new compressive molding technology used in this study was effective in outstanding mechanical strength of 3D-PFTM, the thinnest 0.1 mm commercially available Ti-membranes. In addition, this method provided excellent manageability by prefabricating the membrane in 3D form [13].

Animal studies were conducted to compare the effectiveness of 3D-PFTM with the most widely used barrier membrane by evaluating biocompatibility, clinical manageability, and bone regeneration efficiency using the peri-implant buccal bone defect models in the beagle dogs [33]. The collagen membrane was chosen as the control in this experiment because it has been clinically preferred over Ti-membrane since good biocompatibility and a second operation are not required [9–11]. MS-PFTM with inferior mechanical properties over 3D-PFTM in vitro was not included in the in vivo experiments.

In the in vivo study, the membrane exposure was not observed in both experimental and control groups. In previous studies, the smooth surface of Ti-membrane is less sensitive to bacterial contamination than the spongy architecture of resorbable membrane [45], but the sharp edges or surfaces of Ti-membrane due to manual manipulation trigger mechanical irritation at the mucosal flap and lead to exposure of membrane [27]. The use of 3D-PFTM made with new compressive molding technology can minimize the traditional procedures by hand, which includes trimming, contouring, bending, and fixation and compensated the poor clinical manageability [22]. Moreover, it brings the important advantage to reduce the membrane exposure by reducing mucous membrane irritation [28]. It was confirmed that the biocompatibility of 3D-PFTM was so excellent because no specific immune response was observed [46].

In this study, particulate bone grafts were placed on the buccal bone defects and 3D-PFTM system does not require additional Ti-screw for fixation as the existing surgical procedure [47]. 3D-PFTM was applied to the anchor connected to the implant fixture and fixed with a cover cap. This new approach to place the Ti-membrane strengthens the fixation, minimizes mobility from external stresses, and blocks the leakage of bone graft materials [31]. Moreover, the membrane was easily removed using relatively small sized flap during the second surgery [32].

The result of histological analysis shows that the 3D-PFTM was not absorbed and its semi-dorm shape was maintained. Moreover, greater amount of new bones was formed and more particulate bone graft materials were preserved than the collagen membrane. The results of histometric analyses, including NBA (%), BIC (%), NB-OB (%), and OI-OB (%), were significantly higher in the 3D-PFTM group than in the collagen membrane group (*p* < .001). As a result, it was confirmed that 3D-PFTM having space maintenance ability enhanced mechanical properties which were very effective for bone regeneration compared to collagen membrane.

Various evaluations conducted in this study verified that new mechanically compressive molding technology increased the mechanical properties such as stiffness and fatigue resistance of the 3D-PFTM, thereby improving space maintenance ability and clinical manageability of this membrane. Consequently, 3D-PFTM has proven to be more effective on bone regeneration than the most common barrier membrane used in GBR.

Despite the many advantages of the 3D-PFTM, the fundamental limitation of Ti-membrane, the necessity for second surgery for removal, still remained. Further studies into various bone defect types and thicknesses of membrane are needed to positively utilize the 3D-PFTM system for reconstruction of bone defects from a small aesthetic site to a wide range of vertical or horizontal sites. The numerous membranes used in GBR have different natures and it is important to choose and apply the most effective membrane according to the bone defects [48–53].

## 5. Conclusions

Within the limitations of this study, the results indicate that the application of 3D-PFTM by new mechanically compressive molding technology validated increased mechanical properties and clinical manageability, effectiveness of space maintenance ability, and stabilization of the particulate bone graft materials, and biocompatibility finally led to highly efficient bone regeneration.

## Figures and Tables

**Table 1:** Histometric analysis within the area of interest (*n* = 6; %).

Group		NBA (%)	RBA (%)	BIC (%)	NB-OB (%)	OI-OB (%)
CM	Mean ± SD	4.75 ± 1.16	11.11 ± 5.04	19.84 ± 4.26	27.52 ± 4.61	17.84 ± 4.61
	Median	4.83	9.98	19.60	28.35	20.61
3D-PRTM	Mean ± SD	35.86 ± 2.65	19.35 ± 4.47	61.97 ± 4.03	70.33 ± 4.94	62.00 ± 4.29
	Median	36.03	19.89	60.53	69.10	63.50
^*∗∗∗*^ *p*		<.001	<.001	<.001	<.001	<.001

CM, collagen membrane; 3D-PFTM, three-dimensional preformed Ti-membrane; NBA, new bone area; RBA, remaining graft bone area; BIC, bone-to-implant contact; NB-OB, distance from the upper point of new bone to the old bone; OI-OB, distance from the upper point of the osseointegration to the old bone. The symbol “*∗∗∗*” indicates statistical significance between the two groups (*p* < .001).
